# The Impact of DNA Repair Pathways in Cancer Biology and Therapy

**DOI:** 10.3390/cancers9090126

**Published:** 2017-09-19

**Authors:** Anatoly Nikolaev, Eddy S. Yang

**Affiliations:** Department of Radiation Oncology, UAB Comprehensive Cancer Center, Birmingham, AL 35249, USA; anatolynikolaev@uabmc.edu

Genomic instability is one of the key hallmarks of cancer progression [1]. Evolutionary conserved pathways of DNA repair play a critical role in the maintenance of genomic stability [1,2]. Therefore, it is not surprising that factors responsible for sensing and repair of DNA damage were found to be key regulators of tumorigenesis [3]. In fact, the targeting of DNA repair pathways has come to the forefront as a bona fide therapeutic strategy. This is evident by the emergence of poly (ADP-ribose) polymerase (PARP), DNA dependent protein kinase (DNA-PK), ATM and Rad3 related (ATR), checkpoint kinase-1 (Chk1), and others as potential drug targets for a variety of malignancies, including breast, ovarian, and head and neck cancers [4,5]. In this Special Issue of *Cancers*, the role of DNA repair pathways in cancer therapy, tumor biology, radiation sensitivity, and resistance will be discussed.

Perhaps the most developed targeted DNA repair therapies are the PARP inhibitors, which are synthetically lethal with homologous recombination (HR) repair deficient tumors. These drugs were recently FDA approved for recurrent breast cancer susceptibility gene 1 and 2 (BRCA1/2) mutant epithelial ovarian cancers that are responsive to platinum based therapy. Despite their promise, resistance to PARP inhibition represents a formidable clinical problem [6]. In their article, “Understanding Resistance Mechanisms and Expanding the Therapeutic Utility of PARP Inhibitors”, Lim and Tan reviewed the most recent clinical trials of PARP inhibitors, such as the hallmark phase III NOVA trial leading to FDA approval of niraparib regardless of BRCA1/2 mutation status [7]. This is an exciting area as PARP inhibitors are promising strategies for HR defective breast and prostate cancers. This article also summarized the mechanisms of acquired resistance, such as restoration of the open reading frame in the RAD51 domain of BRCA1, loss of non-homologous end-joining (NHEJ) factor p53BP1, and over-expression of multidrug efflux pumps such as p-glycoprotein (Pgp). The efforts to overcome resistance to PARP inhibitors such as combining them with inhibitors of cell cycle regulators Wee1, ATR and Chk1, or with phosphatidyl-inositol-3 Kinase (PI3K)/AKT/mammalian target of rapamycin (mTOR) pathway inhibitors were also discussed [7].

Another emerging strategy to target the DNA damage/repair pathways is inhibiting the ATR kinase or its downstream target Chk1. These two molecules are believed to activate G2/M and S checkpoints via phosphorylation of Wee1, Cdc25A and Cdc25C and inhibition of CDK1 and CHK2. This pathway is especially important in p53 and Rb mutant tumors which are deficient in G1/S checkpoint [5,8,9,10]. The article “Targeting the ATR–CHK1 Axis in Cancer Therapy” by Rundle et al. reviews the ATR-CHK1 pathway and a number of potent and selective inhibitors of ATR and Chk1. These compounds have demonstrated, in preclinical studies, chemo-sensitization, radiosensitization, and chemo-radio-sensitization of tumor cells lines and human xenograft models. ATR and Chk1 inhibitors are currently in early stages of clinical development for a number of solid tumors [11]. For instance, the ATR inhibitor VX-970 is being tested in combination with chemoradiation in p53 mutant locally advanced head and neck cancers, and in combination with whole brain radiation for NSCLCs with brain metastasis. The CHK1 inhibitor LY2606368 is currently undergoing a phase Ib clinical trial in combination with chemotherapy and IMRT in advanced head and neck tumors and phase II trials as a single agent in solid tumors with replicative stress or HR deficiency, and in triple negative breast cancers and castration-resistant prostate cancers [11].

Non-homologous end-joining is an alternative pathway to homologous recombination in the repair of double strand breaks (DSBs) [12,13]. However, as we learn from the review by Sishc et al., “The Role of the Core Non-Homologous End Joining Factors in Carcinogenesis and Cancer”, over-activation or attenuation of the NHEJ pathway may also lead to carcinogenesis by promoting chromosomal rearrangements such as translocations. Evidence is presented supporting the association between overexpression of NHEJ core factors and tumor aggressiveness, metastatic disease, poor survival, and radio-resistance. Conversely, decreased expression of NHEJ factors, such as DNA-PK, is associated with radiosensitization and improved response to radiation treatment. Several small molecule inhibitors of DNA-PK had been identified and are now being tested as radiosensitizing agents in phase I–II clinical trials. For example, a clinical trial is evaluating the DNA-PK inhibitor MSC2490484A together with either radiation therapy or radiation with cisplatin in head and neck and thoracic malignancies. The authors hypothesize that tumor cells deficient in Fanconi Anemia (FA) or HR DNA repair are addicted to the NHEJ pathway and therefore targeting NHEJ overexpressing tumors may represent a promising approach for sensitization to conventional DNA damaging agents such as ionizing radiation [14].

Ionizing radiation is known to trigger multiple types of DNA damage which if left unrepaired, can ultimately lead to cellular demise. Therefore, it is not surprising that DNA repair pathways are evolutionary conserved in both animal and plant kingdoms [2]. In their article, “Bridging Plant and Human Radiation Response and DNA Repair through an In Silico Approach”, Nikitaki et al. developed a plant-based biological sensor of DNA damage by identifying DNA repair orthologue genes in a model plant Arabidopsis thaliana that can be used as biomarkers of radiation exposure. A subset of the orthologue genes known to be involved in DSB repair, including BRCA1, BARD1 and PARP were further validated experimentally in vitro in the comet DNA repair assays. Such model plant orthologues of human DNA repair genes may be used in the future as a foundation for the design of a plant bio-dosimeter for distinct types of electromagnetic radiation exposure [15].

Carbon ion therapy is a promising radiation therapeutic modality with a number of advantages over the conventional photon therapy [16]. The review, “Carbon Ion Radiotherapy: A Review of Clinical Experiences and Preclinical Research, with an Emphasis on DNA Damage/Repair”, by Mohamad et al., discusses the unique physical and biological characteristics of carbon ion therapy and the results of recent clinical trials of this therapeutic modality [16]. The physical properties of the carbon beam contributing to the proposed superior delivery of radiation as compared to photons include superior linear energy transfer, dose verification by PET imaging, improved dose distribution and lateral focusing due to a spread out Bragg peak, and magnetic steering of charged particles allowing for dose painting. The unique biological properties of the carbon ion therapy are its increased relative biological effectiveness, complex patterns of DNA damage that are difficult to repair, cell cycle independent killing, and its effectiveness under hypoxic conditions due to a relatively low oxygen enhancement ratio. Carbon ion therapy had been evaluated in a number of phase I–II clinical trials with encouraging results for the deep seated, highly aggressive, recurrent tumors located in critical areas. Significant promise has been demonstrated for osteosarcomas and soft tissue sarcomas, head and neck including base of the skull tumors such as chordomas and chondrosarcomas, prostate, cervical and pancreatic cancers, and hepatocellular carcinomas. The phase III clinical trials of carbon ion therapy for skull base chordomas and chondrosarcomas are ongoing [17].

Oxidative stress induced by either ionizing radiation or chemotherapy drugs is another one of the major factors contributing to DNA damage by reactive oxygen species and tumor cell killing [18]. Conversely, cancer cells upregulate a number of stress response pathways aimed at mitigating the deleterious effects of oxidative damage of DNA [19]. In “MTH1 as a Chemotherapeutic Target: The Elephant in the Room”, Samaranayake et al. review the role of the human MutT homologue 1 (MTH1), which is a DNA repair enzyme that removes oxidized purine nucleotide derivatives, as a potential target. MTH1 overexpression in RAS-driven malignancies, such as pancreatic and lung cancer, had been linked to poor survival and increased rates of tumor recurrence [20]. A number of MTH1 inhibitors have been developed and tested in a variety of tumor cell lines with mixed results. The preclinical evaluation of these inhibitors in oxidative stress driven tumor models is ongoing [20].

Cancer stem cells are believed to have superior DNA damage repair capabilities and therefore are able to escape the cell killing effects of conventional chemotherapy and radiation [21,22,23]. The review by Annovazzi et al., “Chemotherapeutic Drugs: DNA Damage and Repair in Glioma”, discusses the role of glioblastoma stem cells in resistance to temozolomide (TMZ), with the major mechanism being overexpression of O(6)-methylguanine-DNA methyltransferase (MGMT). Additional mechanisms contributing to TMZ resistance include acquired mutations in mismatch repair genes and tp53, as well as activation of ataxia telangiectasia mutated (ATM) and ATR [24].

DNA repair pathways are also dysregulated in bladder cancer [25]. In “DNA Repair Pathway Alterations in Bladder Cancer”, Mouw discusses the drivers of genomic instability in high grade muscle invasive bladder cancers, which were reported to have high rates of genomic alterations including copy number variations gene mutations and chromosomal rearrangements [26]. While p53 mutation was most commonly observed in this tumor cohort, a nucleotide excision repair (NER) pathway gene ERCC2 was mutated in a substantial number of cases (~15%). Somatic ERCC2 mutations were found to be associated with increased sensitivity to cisplatin therapy improved survival, and therefore may be predictive of cisplatin response. Low levels of another NER pathway protein, ERCC1, were found to be associated with improved response to cisplatin, presumably because ERCC1 is involved in the removal of bulky DNA adducts caused by platinum-based drugs. Low levels of double strand break (DSB) repair protein MRE11 were shown to be associated with poor outcomes in bladder cancer patients treated with radiation. It was hypothesized that decreased detection of DSB in these patients led to enhanced proliferation of tumor cells. In addition, mutations in other DNA repair genes such as ATM, FANCD2, and BRCA1/2 were found to be associated with improved recurrence-free survival in patients receiving perioperative chemotherapy [26].

In summary, this Special Issue of *Cancers* is a collection of basic, translational, and clinical articles discussing the major impact that DNA damage response and repair pathways is playing in cancer biology and therapy. More work is needed to advance the field with regards to rationally combining therapies targeting DNA repair with other agents, including immunotherapy.
